# Influence of negative stereotype on physical activity level among older adults during a training session

**DOI:** 10.3389/fspor.2022.998724

**Published:** 2022-11-25

**Authors:** Maxime Deshayes, Angèle Palermo, Karim Korchi, Antony G. Philippe

**Affiliations:** UNIV. NIMES, APSY-V, F-30021 Nîmes Cedex 1, France

**Keywords:** stereotype threat, physical activity, inactivity time, older adults, objective and subjective measures

## Abstract

The present research examined the effect of a negative stereotype induction on older adults' physical activity level, measured objectively and subjectively. Twenty older adults (18 women and two men; *M*age = 67.4, *SD*age = 4.4) were assigned to a control condition, a neutral condition and a negative stereotype condition during three separate visits (i.e., within-subject design). In each physical activity session, participants performed the same training. Objective physical activity level was the time spent at moderate to vigorous intensity measured by accelerometry and subjective physical activity level was measured with the RPE-session method. Inactivity time was also objectively assessed. Results revealed no effect of the different conditions on objective physical activity level, but subjective physical activity level and inactivity time were lower in the neutral condition and in the negative stereotype condition compared to the control condition. It was suggested that when a negative stereotype is induced, participants perceived the task as less intense compared to the control condition, which result in less inactivity time, suggesting that the negative stereotype had a positive influence on physical activity. Another interesting result was that effects were similar in the negative stereotype condition and in the neutral condition, revealing that the neutral condition might not be a control condition. While these results are not in line with the stereotype threat literature, they echo previous recent studies also showing a positive effect of a negative stereotype induction, calling into question the stereotype threat theory.

## Introduction

With the increase of age, a decrease in physical activity (PA) participation is mostly observed accompanied by a physical decline [e.g., (1)], which is often considered as an inevitable biological process. However, and without neglecting the biological process involved, some researchers have suggested that psychological factors, especially stereotypes, play also an important role in this reduction [e.g., (2–4)]. Overall, two complementary frameworks have provided some evidence to this assumption: the stereotype embodiment theory (5) and the stereotype threat (ST) theory (6). In the present research, we only focused on the ST theory, which has been few explored when considering aging stereotypes, especially their influence on PA. Specifically, the aim of the study was to investigate the influence of a negative stereotype induction on PA level during a training session.

According to the ST theory, when a negative stereotype toward one group is induced, in a context where this stereotype can be applied, a decreased performance is expected (6). A large number of studies have found and replicated this performance impairment on various domains, on many populations, using different stereotypes [e.g., (7, 8)]. More precisely, concerning older adults, most studies have replicated this deleterious effect in a large variety of domains [for meta-analyses, see (9, 10)]. Indeed, meta-analyses revealed a reliable ST effect on older adults' episodic and working memory (9) and on cognitive performances [e.g., math tests; (10)]. However, to the best of our knowledge, only nine studies have been conducted in the physical domain and results are mixed. For example, it has been shown that inducing a negative stereotype may negatively impact maximal strength and walking speed [e.g., (11–13)]. Conversely, some others studies revealed that these same variables are not impacted during a ST situation, as well as balance, flexibility and endurance (14–18). Finally, a recent study also found a positive effect of a negative stereotype induction during a submaximal strength task (19).

All studies conducted in the physical domain have the common point that they only focused on isolated tasks measuring physical capacities (e.g., strength, balance, walking), and mostly in non-ecological situations. For example, in Marquet et al. (17), participants' physical capacities were assessed in a laboratory with only the experimenter and the participant [see also (13, 18) for examples]. Moreover, as far as we know, there is no information concerning the possible durability of the ST effects immediately after the stereotype induction. A recent study has suggested, in another stereotyped population, that the effects of ST would last at least 20 min (20). A good way to investigate the effects of ST in the physical domain and its durability in an ecological context is the investigation of PA level during a training session, which is currently unknown. This exploration seems necessary for several reasons. Firstly, PA level is an important health-related outcome. Indeed, in older adults, it has been related to cognitive and physical functioning [e.g., (21, 22)], frailty [e.g., (23)], cancer risks [e.g., (24)] and mortality [e.g., (25)]. Moreover, it is well established that a large part of the older adults' population is insufficiently active and does not meet PA guidelines [e.g., (26)]. Thus, a potential diminution of PA level induced by the negative stereotype, would lead to an even greater distance from the recommendations and would therefore be devastating for older adults' health since PA level must reach a threshold to elicit physical adaptations and benefits. If training sessions are too light and/or too distant in time, no physical benefits would be observed over time (27). Therefore, the present research focused on the effects of a negative stereotype induction on PA level measured during a training session.

In the literature, different methods have been used to quantify subjective and objective PA level. A subjective method commonly used is the Session-RPE method (28) based on CR-10 RPE scale (29). Several objective methods have been proposed to quantify PA level such as evolution of blood lactate, heart rate and oxygen consumption (30). For intermittent exercise trainings, these methods are limited because these physiological variables are not constant over time (31). Thus, the use of accelerometry appears to be a good alternative because both PA duration and intensity are recorded over time and allow an objective measure of PA (i.e., moderate to vigorous physical activity: MVPA) as well as an objective measure of inactivity [i.e., inactivity time: IT (32)]. Crossing objective and subjective measures of PA level seems important to have an overview of what participants objectively do during the session but also how they subjectively perceived it. Moreover, past studies have shown that, under ST, results may differ between self-reported measures and objective measures [e.g., (20, 33)].

Previous studies have shown that PA level can be influenced by psychological factors such as personality traits, social comparison, or even stereotypes [e.g., (3, 34, 35)]. For example, Arigo et al. (34) found that when women experienced more comparisons than usual, they were objectively 7 to 14% less active in the following 30 min. Also, Emile et al. (3) have observed in a cross-sectional study that the more older adults hold negative aging stereotypes related to the physical domain, the more their PA level is low. However, no study has attempted to observe the situational and immediate effect of the induction of a negative stereotype on PA level.

Therefore, the main aim of the present research was to observe the effect of a negative stereotype induction on older adults' PA level measured objectively through accelerometry (i.e., MVPA and IT) as well as subjectively using the Session-RPE method. Based on the mixed results observed in the literature, we did not make hypotheses.

## Method

### Participants

Twenty older adults (i.e., 18 women and two men) ranging from 60 to 76 years old (*M*age = 67.4, *SD*age = 4.4) were recruited. A sensitivity power analysis, based on a within-subject design with repeated measures, assuming an α of 0.05 and power of 0.80, indicated that our sample size allows us to detect moderate-to-large effects of *f* = 0.30. Participants were directly contacted during their sports class and provided informed consent. The inclusion criteria included: more than 60 years old, being identified with the physical activity domain, being affiliated to a physical activity group. The exclusion criteria included: any bone, muscle or tendon trauma of the limbs (e.g., osteoarthritis). According to the ST theory (6), individuals need to identify with the domain to be susceptible to ST (36). Consequently, in the present research, we only recruited participants from a physical activity group, as in Chiviacowsky et al. (13) and Deshayes et al. (19). Moreover, to ensure that they were identified with the physical activity domain, participants, prior to the experiment, completed two 7-point items [i.e., “it is important for me to have good physical abilities” and “it is important for me to be good during physical activities,” (20)], with responses ranging from 1 (strongly disagree) to 7 (strongly agree). All the recruited participants had a mean score higher than 4 (*M* = 6.9, *SD* = 0.3) and were consequently added in this study.

### Study design

In the present research, a within-subject design was used because (i) it has greater statistical power (37) and (ii) it enables better observation of individual differences, as each participant is assigned to each condition. This type of design has gained popularity these past few years to investigate ST effects [e.g., (20, 38, 39)], especially in older adults (15). In this study, three visits were included reflecting the three conditions used. All the participants began with the control condition [as in (39)], followed by two randomized conditions: a neutral condition and a negative stereotype condition.

### Materials and measures

Objective PA level and IT were measured with tri-axis accelerometers GT3X (Actigraph, Pensacola, FL, USA). Participants had to wear the accelerometer on the right side of the hip, adjusted with an elastic belt, during all training sessions. Actilife v-6.13.4 Lite Pro software was used to extract PA and IT values. Data were downloaded in 1 second epoch to measure PA and IT. According to Freedson algorithm, we defined IT as 0–99 counts per minute and MVPA > 1,952 per min (40).

Subjective PA level was evaluated according to the Session-RPE method (28). After the session, participants had to report the perceived intensity of the session on a CR-10 RPE scale (29). This value was multiplied by the duration of the session expressed in minutes (28). This method is frequently used to monitor PA level in various populations and expertise level [see (41) for a review].

### Physical activity session

During the three visits, separated by 7 days, participants performed the same physical activity session, lasting ~45–50 min at the same time of day. The session content is provided as Supplementary material to this paper. Specifically, in order to observe how the different conditions influenced PA level, instead of asking participants to perform a certain number of repetitions, participants were requested to do as much as effort as they can in a given time. Participants were divided into groups of 5–10 people as in a previous study (27), which remained the same during the three sessions. The experimenter was the same all along the experimentation and was requested to have the same implication during all sessions and to use the same tone of voice in order to limit intersession variability.

### Procedure

During the first session (i.e., control condition), participants were greeted by a female experimenter. After giving consent, all participants responded individually to the domain identification questionnaire. Prior to the session, each participant was given an accelerometer. The protocol was then initiated and participants performed the PA lesson. At the end of each session, participants reported their perceived intensity. Participants came back to the fitness class 1 week later, were randomly assigned to another condition (i.e., negative stereotype condition or neutral condition) and realized the same training lesson. Then, participants came back a last time to perform the final training session 1 week later in the condition to which they had not yet been assigned (i.e., negative stereotype condition or neutral condition). During each session, as a stereotype manipulation check, participants were asked: “For this training session, your PA level will be compared to:” with three answers options: “younger people,” “people of the same age” or “older people.” All participants correctly answered the question in each session. Finally, they were thanked and debriefed.

### Stereotype manipulations

Given the within-subject nature of the design, we did not activate stereotypes by manipulating task diagnosticity, as is classically done in ST research [e.g., (42)]. Indeed, it was considered that it was not credible to present to the same participants the task as diagnostic of one ability in one condition, and as non-diagnostic of the same ability in another condition (15). Thus, in the negative stereotype condition, the stereotype was activated by telling participants that the goal of the study was to examine differences in PA level between older adults and younger adults and that their PA level would be compared to younger adults. In the neutral condition, participants were told that the goal of the study was to examine differences in PA level among older adults aged 65 years and over and that their level of physical activity would be compared to adults of the same age. They were further informed that older adults referred to people aged 65 years and over (i.e., in the neutral condition), and that younger adults referred to people aged 30 years and less (i.e., in the negative stereotype condition). This manipulation has been recently used to evaluate the effect of stereotype in older adults during a physical task [(15), see also (12)]. In the control condition, nothing was said to participants [e.g., (43)].

### Data analysis

#### Main analysis

Variable data normality was checked using the Shapiro–Wilk test and homogeneity of variance with the *F*-test. Since normality was assumed (*ps* > 0.05) for objective PA level and IT, a one-way ANOVA with repeated measures was conducted with Conditions (i.e., control *vs*. neutral *vs*. negative stereotype) as a within-subject factor. Paired sample *t*-tests were used for comparison within groups and Bonferroni correction was used to adjust for multiple comparisons. For subjective PA level, normality was not assumed (*ps* < 0.05). A non-parametric Friedman ANOVA was thus conducted and Conover's *post-hoc* tests were used for comparisons within groups. Effect sizes were expressed as the eta-squared (i.e., η^2^) for ANOVA analysis, as Cohen d (i.e., *d*) for *t*-tests and as Kendall's W index (i.e., *W*) for Friedman ANOVA.

#### Complementary analysis

To the best of our knowledge, no study has investigated the durability of the effect of the stereotype immediately after its induction. In other words, does the stereotype influence participants during the entire session or does its effect dissipate over time? Therefore, each session was also split into two times: T1 corresponding to the first 50% of the training session and T2 referring to the last 50% of the training session and a 3 (Conditions) x 2 (Time of measurement) ANOVA was conducted with Conditions and Time of measurement as within-subject factors.

## Results

Means and *SD*s for the study variables are presented in Table 1.

**Table 1:** Means (±standard deviations) of the dependent variables of the study.

	**Conditions**					
**Variables**	**Negative stereotype**		**Neutral**		**Control**	
MVPA	20.8 ± 6.0		21.0 ± 5.6		20.5 ± 5.9	
IT	12.9 ± 2.1^*^		13.5 ± 2.4^*^		15.0 ± 3.1	
RPE-Session	184.4 ± 56.3^*^		198.8 ± 36.8^*^		225.2 ± 55.6	
	**Negative stereotype**		**Neutral**		**Control**	
	**T1**	**T2**	**T1**	**T2**	**T1**	**T2**
MVPA	9.4 ± 2.8	11.4 ± 3.4	9.2 ± 2.6	11.8 ± 3.3	9.3 ± 3.0	11.2 ± 3.1
IT	7.0 ± 1.5^*^	6.0 ± 1.0	7.5 ± 1.3^*^	6.0 ± 1.3	8.5 ± 1.6	6.5 ± 1.8

MVPA, moderate to vigorous physical activity; IT, inactivity time; RPE, rate of perceived exertion.

* p < 0.05 indicated a significant difference with the control condition.

### Main analysis

**Figure 1:**
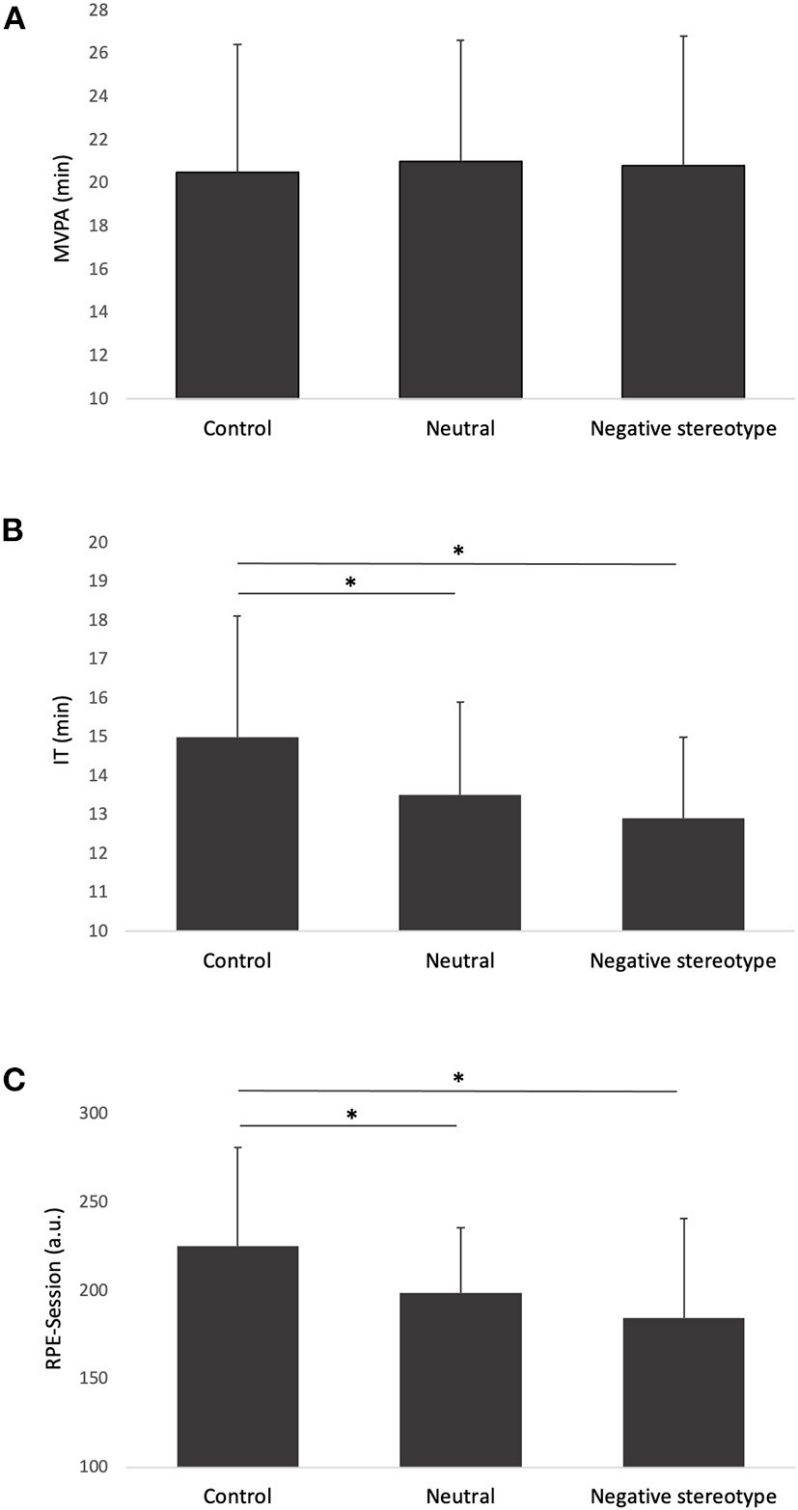
Difference of MVPA **(A)**, IT **(B)**, and RPE-Session **(C)** between the three different conditions.

For objective PA level, the ANOVA revealed no Condition main effect (*F*_(2, 36)_ = 0.65; *p* = 0.53; η^2^ = 0.04), suggesting that the different conditions had no effect on PA level (see Figure 1A). For IT, results showed a Condition main effect (*F*_(2, 36)_ = 9.29; *p* < 0.001; η^2^ = 0.34). Specifically, IT was higher in the control condition (*M* = 15.04; *SD* = 3.13) compared to the neutral condition (*M* = 13.49; SD = 2.36; *p* = 0.01; *d* = 0.70) and to the negative stereotype condition (*M* = 12.94; SD = 2.12; *p* < 0.001; *d* = 0.95). There was no significant difference in IT between the neutral condition and the negative stereotype condition (*p* = 0.84; *d* = 0.25; see Figure 1B).

For subjective PA level, a Condition main effect emerged (χ2 _(2)_ = 17.23; *p* < 0.001; Kendall's *W* = 0.58). Subjective PA level was higher in the control condition (*M* = 225.15; *SD* = 55.62) compared to the neutral condition (*M* = 198.80; *SD* = 35.75; *p* = 0.008) and to the negative stereotype condition (*M* = 184.40; *SD* = 56.26; *p* < 0.001). There was no significant difference in IT between the neutral condition and the negative stereotype condition (*p* = 0.21; see Figure 1C).

### Complementary analysis

For objective PA level, the ANOVA revealed no Condition main effect (*F*_(2, 36)_ = 0.65; *p* = 0.53; η^2^ = 0.04) but a Time of measurement main effect (*F*_(1, 18)_ = 37.47; *p* < 0.001; η2 = 0.68). More importantly, there was no significant Condition x Time of measurement main effect (*F*_(2, 36)_ = 1.51; *p* = 0.24; η^2^ = 0.08).

For IT, results revealed a Condition main effect (*F*_(2, 36)_ = 9.29; *p* < 0.001; η^2^ = 0.34), a Time of measurement main effect (*F*_(1, 18)_ = 69.11; *p* < 0.001; η^2^ = 0.79), as well as a Condition x Time of measurement interaction effect (*F*_(2, 36)_ = 3.3; *p* = 0.05; η^2^ = 0.16). Specifically, at T1, participants' IT was higher in the control condition (*M* = 8.54; *SD* = 1.64) compared to the neutral condition (*M* = 7.51; *SD* = 1.34; *p* = 0.03) and to the negative stereotype condition (*M* = 6.96; *SD* = 1.48; *p* < 0.001). There was no significant difference in IT between the neutral condition and the negative stereotype condition (*p* = 1.0). At T2, there was no significant difference between the three conditions concerning IT (all *p*s > 0.05). This complementary analysis revealed that the effects on IT were present only during the first part of the session.

## Discussion

The aim of the present study was to observe the effect of a negative stereotype induction on older adults' objective and subjective PA level and IT during a training session. Results revealed no effect of the different conditions on objective PA level (i.e., MVPA), but subjective PA level (i.e., Session RPE) and IT were lower in the neutral condition and in the negative stereotype condition compared to the control condition. Overall, these results are not in line with the ST theory and are worthy of discussion.

Firstly, as evoked, there was no significant difference between control and negative stereotype conditions concerning objective PA level. In other words, the time spent at moderate to vigorous PA intensities was similar between the two conditions. Even if this result is not in line with the ST theory, it seems however in line with most recent studies conducted in older adults showing no effect of the negative stereotype induction on objective variables in the physical domain [e.g., (15, 16, 18)]. Chiviacowsky et al. (13) have suggested that the absence of effect of the induction of a negative stereotype on physical performance could be due to a delayed effect. According to them, participants under ST would adopt a prevention-focus approach [i.e., focus on avoiding failure, (44)]. Adopting this approach can annihilate the ST effect in a short term, due to resource involvement but not in a long term due to cognitive exhaustion. Supporting this assumption, Chiviacowsky et al. (13) did not found any ST effect immediately after the induction of the negative stereotype, but an effect the day after. All of these results revealed that negative stereotypes had a minor influence on objective measures in the physical domain in older adults, immediately after their inductions.

However, subjective PA level was impacted. Subjective participants' PA level was significantly lower in the negative stereotype condition compared to the control condition. This result suggests that participants in the negative stereotype condition felt that their PA level was lower even if it was objectively similar to the control condition. This mixed result for the same variable measured with different tools is not that surprising. Indeed, past studies have also shown that the effects of negative stereotypes could be different according to the tools used [e.g., (20, 33)]. For example, Deshayes et al. (20) investigated the effect of the induction of a negative stereotype on task involvement, in another population. Interestingly, they found, as in the present research, different results between objective and subjective measures. Also, this result is in line with recent studies suggesting that even if no effect is observed on an objective physical variable, the negative stereotype can however have an effect on psychological variables [e.g., (18, 45)]. For example, Deshayes et al. (18) found that the negative stereotype induction did not influence older adults' endurance performance but impacted negatively subjective age. In the same vein, Chalabaev et al. (15) found that negative stereotype induction does not influence walking speed but increases cognitive load.

Subjective PA level was measured with the “Rate of Perceived Exertion” (i.e., CR 1-10 RPE value) and previous studies have found that this variable can be influenced by a ST situation [e.g., (20, 38)]. Gray et al. (38) found during a fatiguing intermittent task, that RPE increased slowly when a negative stereotype was induced [i.e., positive effect; see also (20)]. As a training session is based on intermittent exercises and may be fatiguing, the result observed in the present research is in line with the study conducted by Gray and Colleagues. However, how the lowest subjective PA level observed in the present research in the negative stereotype condition can be interpreted? Two opposing hypotheses may be outlined: Did participants felt they have been less active because (1) they perceived the task as less intense or (2) they were less motivated? The combination of the subjective PA measurement and the result observed on IT give credibility to the first hypothesis. Indeed, in order to assess the subjective PA level, participants were requested to answer the following question: “How was your session?” This instruction is closely related to task intensity since a close association has been observed between RPE and task intensity in older adults (46). Moreover, we observed that participants in the negative stereotype condition were less inactive than participants in the control condition. Therefore, we may suggest that in the negative stereotype condition, participants perceived the task as less intense than under the control condition, which result in less IT. In addition, it can be noted that the effect of the stereotype on IT seemed to dissipate during the session. Indeed, participants were less inactive in the negative stereotype condition compared to the control condition only during the first 50% of the session (i.e., ~25 min). To the best of our knowledge, this study is the first showing that the negative stereotype is effective during a short period before quickly dissipating.

In sum, we found no effect on objective PA level but a positive effect on subjective PA level and on objective IT. These positive results echo previous recent studies also showing a positive effect of the induction of a negative stereotype, calling into question the ST theory [e.g., (19, 20, 38, 43, 47)]. If individuals have the feeling of being less active whereas objective PA level remains the same, the negative stereotype induction seems positive. Indeed, one of the most cited barriers to PA is the feeling of fatigue, related to the perceived exertion [i.e., they do not engage in PA because they perceive it as too intense; (48)]. Thus, if PA is perceived as less intense, individuals could engage more in it. However, does this mean that we should threaten older adults to increase their engagement in PA? Based on the stereotype spill-over theory (49), in addition to influencing performance of the stereotyped domain, inducing a negative stereotype can spill over and impact diverse array of non-stereotyped domains [e.g., negative stereotypes toward older adults' physical capacities have a negative influence on cognitive load; (15)]. Therefore, using negative stereotypes seems dangerous.

A last interesting result is that effects observed in the negative stereotype condition were also found in the neutral condition. Specifically, even if objective PA level was not affected, participants in the neutral condition had a lower subjective PA level and were less inactive than in the control condition. These results suggest that the neutral condition may be not a control condition. Some previous studies conducted among older adults investigated the effects of ST by only comparing the neutral condition and the negative stereotype condition (12, 15). Chalabaev et al. (15) did not find any difference between these two same conditions on physical performance. It may be possible that these two conditions influenced physical performance but that this effect was not detected because of the lack of a real control condition. Therefore, it appears necessary to use a control condition when investigating ST among older adults and future studies should focus on the impact of this neutral condition in the physical domain.

Despite interesting results, the current study presents some limits and perspectives. Firstly, even if the sample size is consistent with previous studies conducted in older adults [e.g., (13, 19)], it could be interesting in further research to include more participants. This would allow to increase the statistical power, detecting smaller effect and examine the mechanisms involved in the observed modifications. Secondly, the task used in the present research was not performed at maximal performance as it is classically done in the ST literature [e.g., maximal strength; (12)]. Other recent studies using submaximal tasks did also not find the negative effect expected by the negative stereotype induction [e.g., walking at a self-paced speed, (15); performing submaximal contractions, (19)]. Therefore, it is possible that during submaximal tasks, the induction of a negative stereotype does not have a negative influence, even a positive, in older adults in the physical domain. Thirdly, subjective PA level was only recorded at the end of the session as classically done (50). It would have been interesting to have an intermediate value to examine if the ST operate on subjective PA level during the entire session or, as for IT, during only the first half. Fourthly, recruited participants were highly identified with the PA domain, which is recommended when ST is investigating. However, it could be interesting to replicate this study with community-dwelling older adults, not necessarily identified with the PA domain, in order to generalize, or not, this effect, in the general population. Finally, our stereotype manipulations were chosen to fit with the design of the study [see also (15)]. However, it could be interesting to replicate this study with a between-subject design with a stereotype manipulation based on task diagnosticity, as it was also used in ST research [e.g., (42)].

To conclude, the present research found that a negative stereotype induction had no influence on objective PA level but have a positive effect on IT and subjective PA level during a training session, which is contrary to the ST theory. Moreover, it appears necessary in future research to add a control condition when investigating ST since the neutral condition seems not to be a control condition.

## Data availability statement

The raw data supporting the conclusions of this article will be made available by the authors, without undue reservation.

## Ethics statement

Ethical review and approval was not required for the study on human participants in accordance with the local legislation and institutional requirements. The patients/participants provided their written informed consent to participate in this study.

## Author contributions

MD: conceptualization, methodology, formal analysis, writing—original draft, supervision, and data curation. AP: conceptualization, methodology, and investigation. KK: methodology and formal analysis. AGP: conceptualization, methodology, formal analysis, and writing—original draft. All authors contributed to the article and approved the submitted version.

## Conflict of interest

The authors declare that the research was conducted in the absence of any commercial or financial relationships that could be construed as a potential conflict of interest.

## Publisher's note

All claims expressed in this article are solely those of the authors and do not necessarily represent those of their affiliated organizations, or those of the publisher, the editors and the reviewers. Any product that may be evaluated in this article, or claim that may be made by its manufacturer, is not guaranteed or endorsed by the publisher.
