# Supplementary material for: Influence of negative stereotype on physical activity level among older adults during a training session
Source: Front Sports Act Living. 2022 Nov 25;4:998724. doi: 10.3389/fspor.2022.998724 (PMC9732565; doi:10.3389/fspor.2022.998724)
Supplement: Supplementary file 1 [file Table_1.DOCX]

**Training session**

| Warm-up | Zumba style choreography based on lateral and pelvic movements  Repetition: 3 times  Total duration: 10 to 12 minutes |
| --- | --- |
| Training session content | **Circuit 1**  **Objective:** complete the circuit below as many times as possible in 5 minutes.  - 20 squats + 10 squats pulses  - 20 high-stepping (with or without jump)  - 20 meters of walking lunges  Duration: 5 minutes  Resting time: 2 minutes  **Circuit 2**  - 30 sec of walking/running + 30 sec of crab-walk  - 45 sec of walking/running + 45 sec of crab-walk  - 60 sec of walking/running + 60 sec of crab-walk  Duration: 4 minutes and 30 seconds  Resting time: 2 minutes  **Circuit 3**  **Objective:** complete the circuit below as many times as possible in 5 minutes  20 squats + 10 squats pulses  20 high-stepping (with or without jump)  20 meters of walking lunges  Duration: 5 minutes  Resting time: 2 minutes  **Circuit 4**  - 30 sec of walking/running + 30 sec of crab-walk  - 45 sec of walking/running + 45 sec of crab-walk  - 60 sec of walking/running + 60 sec of crab-walk  Duration: 4 minutes and 30 secondes  Resting time: 2 minutes  **Circuit 5**  **Objective:** complete the circuit below as many times as possible in 5 minutes  20 squats + 10 squats pulses  20 high-stepping (with or without jump)  20 meters of walking lunges  Duration: 5 minutes |
| Cool-down | Duration: 5 minutes (MVPA and IT were not measured during the cool-down). |
